# Breaking limits: rapid sea-to-summit ascent of Mount Everest (8,848 m) in 3 days 23 hours following normobaric-hypoxic pre-acclimatization and staged supplemental oxygen in a non-elite athlete

**DOI:** 10.3389/fphys.2026.1873293

**Published:** 2026-10-12

**Authors:** Irina Zelenkova

**Affiliations:** 1 Sports Equity Lab, Stanford University, Palo Alto, CA, United States; 2 OWNTOP Human Performance Center, Barcelona, Spain

**Keywords:** accelerated ascent, acute mountain sickness, case study, Lake Louise Score, Mount Everest, normobaric hypoxia, pre-acclimatization, supplemental oxygen

## Abstract

**Introduction:**

Traditional ascent of Mount Everest (8,848 m) typically requires 40–50 days of on-mountain acclimatization. Normobaric hypoxia (NH) pre-acclimatization is a practical home-based alternative; however, no peer-reviewed case report has described a sea-to-summit Everest ascent completed in under four days by a non-elite individual without prior on-mountain acclimatization rotations.

**Methods:**

A 40-year-old male recreational mountaineer (baseline VO2max 40.8 ml·kg−1·min−1; BMI 30.1 kg·m−2) completed an 18-week structured NH pre-acclimatization program comprising 48 nights of hypoxic sleep (~377 h at simulated altitudes of 1,850–5,486 m; FiO2 16.7–10.5%) and 16 hypoxic exercise sessions (34 h at 3,085–6,000 m; FiO2 14.3–9.8%). Hypoxic dose was calculated as Σ[(95/SpO2 − 1) × h × 100]. During the climb, peripheral oxygen saturation (SpO2) and heart rate (HR) were recorded at each camp, and acute mountain sickness (AMS) was assessed using the 2018 Lake Louise Score (LLS). Supplemental oxygen (O2) was initiated at 0.5 L·min−1 at the start of the ascent from Everest Base Camp (EBC, 5,360 m) and titrated to SpO2 and symptoms up to 3.0 L·min−1 during the summit push.

**Results:**

The program yielded a combined hypoxic exposure of 411 h and a cumulative hypoxic dose of 4,779%·h (sleep 4,353%·h; exercise 426%·h). VO2max increased from 40.8 to 51.0 ml·kg−1·min−1, and body mass decreased from 118.1 to 101.5 kg. The subject departed New York at 00:30 local time (EDT; 10:15 Nepal Standard Time, NST) on 15 May 2025 and reached the summit at 09:22 NST on 19 May 2025, a total sea-to-summit time of 3 days, 23 hours and 7 minutes; the ascent from EBC to the summit took 60 hours and 22 minutes. SpO2 was 75% at Camp 3 (7,100 m; HR 95 bpm) and 70% on arrival at Camp 4 (7,950 m; HR 105 bpm) and at the summit (HR 105 bpm). On descent, SpO2 recovered to 80% at Camp 4 and 85% at Camp 2 (6,750 m; HR 110 bpm), and was 85% on room air on return to EBC (HR 62 bpm). No AMS was diagnosed during the ascent (LLS 0–1 at all ascending checkpoints); mild AMS criteria were met on descent (LLS = 3), resolving by return to EBC. No high-altitude cerebral or pulmonary edema occurred.

**Discussion:**

To our knowledge, this case documents the fastest recorded sea-to-summit ascent of Mount Everest by a non-professional athlete. The combination of a structured 18-week NH pre-acclimatization program, objective physiological monitoring, and staged supplemental oxygen, together with the absence of severe altitude-related complications, suggests that rapid ascent without traditional on-mountain acclimatization may be feasible in a well-prepared, closely supervised non-elite individual. Controlled prospective studies are required to quantify the relative contributions of hematological, ventilatory, and mitochondrial adaptations induced by high-dose NH exposure.

## Introduction

High-altitude mountaineering above 8,000 m imposes severe physiological demands through hypobaric hypoxia, requiring progressive acclimatization to avoid acute mountain sickness (AMS), high-altitude cerebral oedema (HACE), and high-altitude pulmonary oedema (HAPE) (Luks et al., 2017; West, 2012). The conventional Everest expedition occupies 40–50 days of on-mountain acclimatization involving repeated excursions to higher camps to stimulate erythropoiesis, increased hypoxic ventilatory response (HVR), and cardiovascular adaptation (Bärtsch and Saltin, 2008).

Normobaric hypoxia (NH) pre-acclimatization achieved at home via hypoxic tents and mask systems provides a practical means of inducing hypoxic adaptation without prolonged high-altitude residency (Muza, 2007; Tannheimer and Lechner, 2020). Although NH is not a complete surrogate for hypobaric hypoxia (Millet et al., 2012), clinical pre-acclimatization benefits have been documented at sufficient NH doses (Fulco et al., 2013). Commercial ‘FLASH’ expeditions using ~300 h of NH sleep at simulated altitudes up to 7,100 m, combined with high-flow supplemental O_2_, have achieved 100% summit rates on Everest, but still last approximately 20 days and require on-site acclimatization (repeated ascents between camps on the mountain) (Tannheimer and Lechner, 2020). The best-documented rapid ascent using NH remains that of elite athlete Kilian Jornet, who climbed Everest twice in one week in 2017 (fastest known time 26.5 h from Rongbuk monastery) following ~260 h of NH sleep and 30 h of high-intensity NH exercise, supplemented by an alpine hypobaric acclimatization phase (Millet and Jornet, 2019).

The fastest documented sea-to-summit ascent of Everest by a non-professional prior to the present case was approximately 12 days by Roxanne Vogel in 2019 (summit reached on day 12 of a 14-day round trip, via the north side) (Vogel, 2019), using a hypoxic tent and altitude chamber protocol. No peer-reviewed case report has described a sea-to-summit time under four days in a non-elite athlete without mountain acclimatization rotations and without pharmacological aids beyond supplemental oxygen. Critically, previous reports have lacked objective physiological data (VO_2_max, body composition, training load metrics, camp-by-camp SpO_2_/HR) documenting the pre-acclimatization response and the in-ascent physiology. The present case provides these data. Large-cohort evidence from Zhao et al. (2024) demonstrates that rapid air travel ascent to 3,650 m is a significant risk factor for AMS (odds ratio [OR] 1.52, p = 0.006) but transitions to a protective factor upon further ascent to 4,400 m, suggesting that early hypoxic stress may prime subsequent adaptation. The aim of this case report is thus to describe the structured 18-week NH program and to report objective physiological data from an acclimatization-free Everest ascent in a well-prepared non-elite individual.

## Case description

This report showcases a descriptive study of a recreational mountaineer during the 18-week structured preparation for sea-to-summit acclimatization-free Everest ascent. The subject was a 40-year-old recreational mountaineer (height 198 cm, body mass 118.1 kg, VO2max 40.8 ml·kg−1·min−1, BMI 30.1 kg·m−2) with no professional or competitive sporting background and no income derived from athletic activity. Using the Participant Classification Framework (McKay et al., 2022), he was classified as Tier 1–2 (Recreationally Active/Trained-Developmental). Training history: the subject began mountaineering in 2020 and had completed eight significant expeditions by 2025, including Ojos del Salado (6,893 m, 2020), Denali (6,190 m, Alaska), Mount Aconcagua (6,962 m, Argentina, January 2024, ascended in 4 days from New York as a proof-of-concept test), and a near-summit Everest attempt in May 2024 (reaching 8,550 m before recurring vision loss required descent). In March 2025, two months before the expedition, the subject sustained four fractures (two in the right arm, two in the right shoulder blade) in an avalanche on Cotopaxi (Ecuador). Medical clearance for the expedition was granted by his supervising physician. All physiological, training, and expedition data reported here were acquired in the course of routine, individualized coaching and medical supervision of the subject’s preparation and ascent, and not as part of a prospectively designed research study; the data were compiled retrospectively into this single-subject case report after the expedition. Formal ethics committee approval was therefore not required in accordance with regulations applicable to single-patient case reports. The study was conducted in accordance with the Declaration of Helsinki, and the subject provided written informed consent for all interventions, data collection, and physiological testing, and for the publication of identifiable expedition details, health-related and physiological data, and performance-related information. Medical supervision was provided by the author throughout all preparation and expedition phases. Full medical screening with standard electrocardiography (ECG), ultrasound, stress test, and full blood test was performed before the preparation period. Full blood-test screening was conducted at the start of the training period, and before and immediately after the expedition. At baseline, iron status was sufficient (serum iron 16.55 µmol·L^−1^, ferritin 152 ng·mL^−1^, transferrin 2.7 g·L^−1^). On the pre-expedition assessment, iron status remained adequate (serum iron 13.6 µmol·L^−1^, transferrin saturation 20%, ferritin 223 ng·mL^−1^), confirming sufficient iron substrate for the erythropoietic demand of the ascent. Markers of fatigue and training tolerance were reassuring throughout: cortisol (436 nmol·L^−1^), total testosterone (453 ng·dL^−1^), hs-CRP (0.62 mg·L^−1^), hepatic and renal function, were all within normal limits, with no biochemical evidence of overtraining.

Cardiovascular screening (12-lead ECG, transthoracic echocardiography, and a maximal exercise stress test) was performed before the preparation period to exclude occult cardiac disease and to confirm the subject’s capacity to safely sustain maximal exertion under severe hypobaric hypoxia. All findings were within normal limits, with no evidence of structural or ischemic abnormality, confirming fitness to proceed. Throughout the preparation period, the subject followed a nutrition plan tailored to meet individual energy and micronutrient requirements, ensuring the prevention of deficiencies.

## Training intervention and measured variables

All training and pre-acclimatization protocols were designed and medically supervised by the author using a patented methodology. The structured preparation phase, inclusive of the final taper, lasted 18 weeks (6 January – 14 May 2025), the taper comprised the final two weeks (28 April – 14 May 2025) before departure. Maximal aerobic capacity (VO_2_max) was assessed with a maximal incremental treadmill test with breath-by-breath gas analysis. The test began at 4.8 km·h^−1^ at 0% grade; speed was increased by 1.6 km·h^−1^ each stage to 11.3 km·h^−1^ at 0% grade, after which grade was increased by 2% each stage at a constant 11.3 km·h^−1^ until volitional exhaustion, using 1-min stages. The program aimed to achieve three primary physiological adaptations: (1) reduction of body mass while preserving lean muscle mass, (2) improvement of cardiorespiratory fitness and neuromuscular efficiency, and (3) enhancement of hypoxic tolerance through targeted pre-acclimatization strategies. All training sessions were documented using TrainingPeaks Software (Louisville, USA) and a MARQ Adventurer (Garmin, USA) heart rate monitor (Garmin, USA). Training load was calculated from the training volume (in h) and with a dedicated compound score [i.e., Training Stress Score TSS ^®^]. Briefly, the TSS is a way to express workload numerically from a single training session with the following formula TSS (a.u.) = (duration (s) x Intensity factor x Normalized power)/(Functional threshold power × 3,600) × 100; where the functional threshold power (FTP) represent the highest average power/pace maintained during 60 min (in a previous training session or race). For sessions performed without pace or power data including hypoxic treadmill and StairMaster sessions in which absolute pace does not reflect physiological effort under reduced FiO_2_ TrainingPeaks computes the Heart Rate Training Stress Score (hrTSS). hrTSS is derived from time spent in each heart rate zone relative to the lactate threshold heart rate, and approximates the physiological load of a session independent of external output metrics. This approach is particularly appropriate for hypoxic exercise, where equivalent external work at altitude produces substantially higher cardiovascular strain than at sea level.

During the preparation phase, the subject performed indoor training under (NH) conditions using a treadmill or StairMaster, selected according to session objective rather than interchangeably. Treadmill sessions (2–3 per week) targeted aerobic conditioning and running economy, whereas StairMaster sessions (2 per week) targeted ascent-specific muscular endurance, replicating the vertical, predominantly concentric locomotion of climbing (quadriceps- and hip-extensor-dominant) with reduced eccentric loading and joint impact. Hypoxic exercise was performed using a mask system connected to a hypoxic generator (Hypoxico, New York, NY), and the subject slept in a hypoxic tent supplied by the same generator system (Hypoxico, New York, NY). Heart rate (HR) and finger peripheral oxygen saturation (SpO_2_) were monitored during NH training and sleep using an oxygen monitor (Choicemmed MD 300 C318, Beijing Choice Electronic Tech Co., Ltd, Beijing, People’s Republic of China and Wellue O2ring Pulse Oximeter, China). Data on hypoxic generator programs, time spent in hypoxia, sleep quality, and oxygen concentration in the tent (measured with a Handi Oxygen Monitor, Salt Lake City, USA) were systematically recorded in an Excel spreadsheet. Training volumes, content, and NH exposure duration were calculated using TrainingPeaks Software and the Excel dataset. During the ascent, data were collected on time in motion, altitude, SpO_2_, HR, heart rate variability (HRV), Lake Louise scores, oxygen flux supply, sleep hours, sleep quality, and subjective feelings (RPE [CR-10] scale). Time and altitude metrics were derived from the subject’s GPS data. For the purposes of this report, “sea-to-summit” time is defined as the total elapsed time from departure at the subject’s sea-level city of residence (New York) to arrival at the summit (8,848 m). The New York departure is used because it represents the subject’s true sea-level point of origin and reflects the real-world, door-to-summit time relevant to non-professional climbers with professional and family commitments; the transfer from Kathmandu to Everest Base Camp included a helicopter leg.

The altitude exposure was recommended using NH passive, intermittent, and active training (prolonged exposure, from 60 min to days; medium duration, 40–60 min) with low, medium, and high intensity with hypoxic dose quantification and an individualized hypoxic training patented protocol elaborated by the author (Vinogradov and Zelenkova, 2020; Zelenkova et al., 2024). Two broad families of hypoxic-dose metrics have been proposed: altitude-based indices such as “kilometer hours” (km·h; the product of simulated altitude and exposure duration), which quantify the external stimulus (Garvican-Lewis et al., 2016), and saturation-based indices such as “saturation hours” (%·h), which quantify the internal physiological stimulus from the degree of arterial desaturation (Millet et al., 2016). Because the physiological response to a given altitude varies substantially with the curvilinear oxyhemoglobin dissociation relationship, training background, prior hypoxic experience, and the type of hypoxia, we used a saturation-based index rather than an altitude-based one. The metric applied here, Σ[(95/SpO_2_ − 1) × h × 100], is of the same form as the “saturation hours” proposed by Millet et al. (2016) [%·h = (98/s − 1) × h × 100], adapted with a 95% reference saturation consistent with our previous dose–response work (Vinogradov and Zelenkova, 2020; Zelenkova et al., 2024). For each session, the SpO_2_ entered into the dose calculation was the mean value from continuous pulse oximetry (e.g., 2,000 m; 1 h at a heart rate of ~121 bpm, corresponding to SpO_2_ 85–89%), progressively increasing in altitude and intensity.

The subject combined sleeping and daily rest in NH conditions within a hypoxic tent, progressively increasing altitudes (altitude range: 1,850–5,486 m). Training began with low-intensity exercise (e.g., 2,000 m; 1 hour at a heart rate of ~121 bpm, corresponding to SpO2 85–89%), progressively increasing in altitude and intensity. The hypoxic training sessions were conducted on a treadmill and StairMaster, under NH conditions, at simulated altitudes ranging from 2,000 to 6,000 m. The relative intensity of aerobic running sessions in altitude was set at 65–70% of VO_2_max, while intensity for long runs was 75-85% of VO_2_max, 85–95% for middle-distance runs and intervals of 2–5 minutes, and 95–100% for high-intensity short intervals. Weekly training load and intensity are presented in **Figures 1**, **2**. Across the preparation period the subject averaged 303 TSS·week^−1^ at a mean intensity factor of 0.50. Training load followed a periodized pattern: an early build to a single peak of 530 TSS in the week of 24 February, recovery weeks in early–mid March (130–155 TSS), and a sustained high-load block (500 TSS·week^−1^) in late March–early April. Notably, the highest-volume week (16 h, week commencing 10 March; **Figure 2**) coincided with only moderate load (325 TSS), reflecting predominantly low-intensity (Z1–Z2) accumulation, whereas peak load weeks combined higher intensity with substantial volume. During the two-week taper (28 April–14 May), weekly TSS was reduced from 378 to 102 AU (73%) while session frequency and brief high-intensity stimuli were maintained, yielding the pre-departure freshness described above. The program also included supervised nutritional guidance, supplementation (no doping agents), and psychological preparation. Pharmacological acclimatization agents (acetazolamide, xenon, dexamethasone) were excluded from prophylactic use throughout.

**Figure 1:**
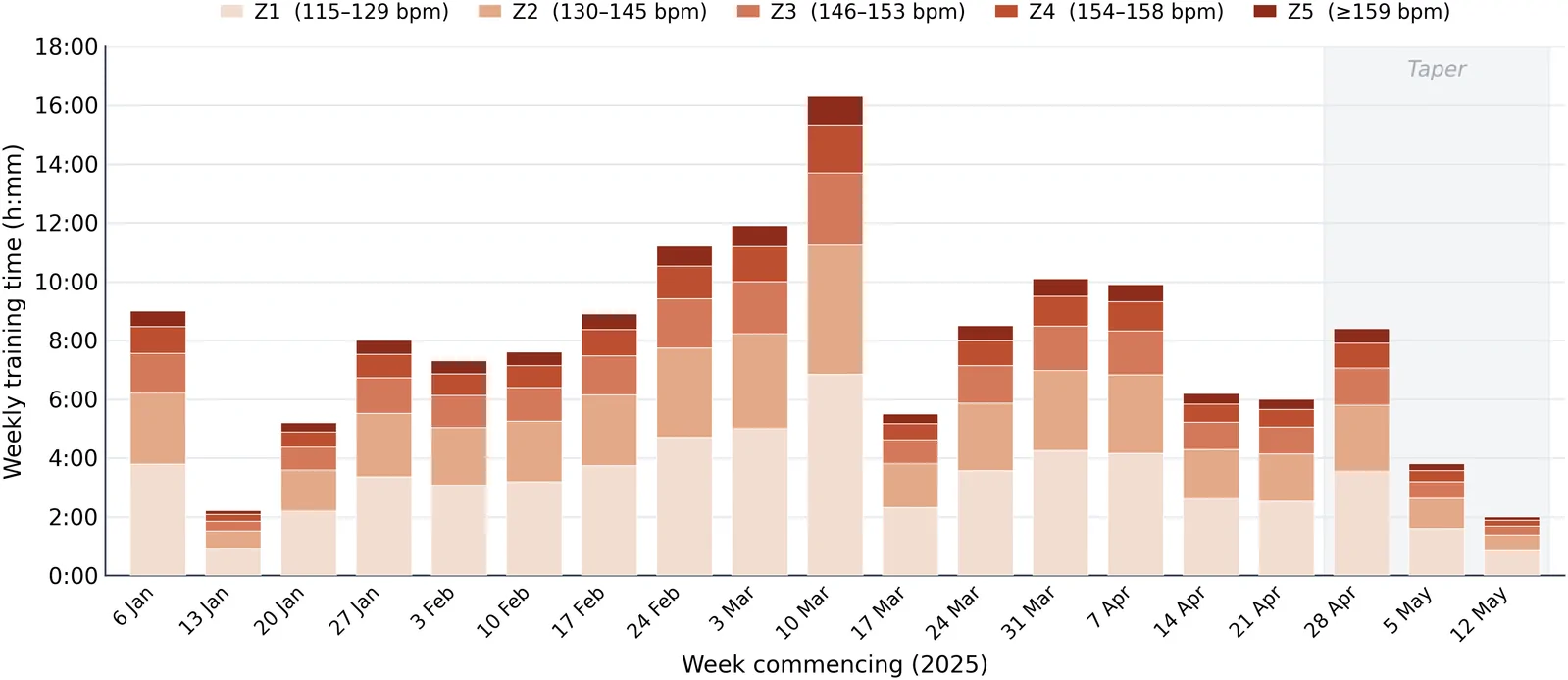
Weekly training time in heart-rate zones across the 18-week preparation period (week commencing 6 January – 12 May 2025), pooled across all session types (running, strength, StairMaster, inclined treadmill). Zones are defined by heart rate: Z1 115–129, Z2 130–145, Z3 146–153, Z4 154–158, Z5 ≥ 159 bpm. The shaded region denotes the two-week pre-departure taper.

**Figure 2:**
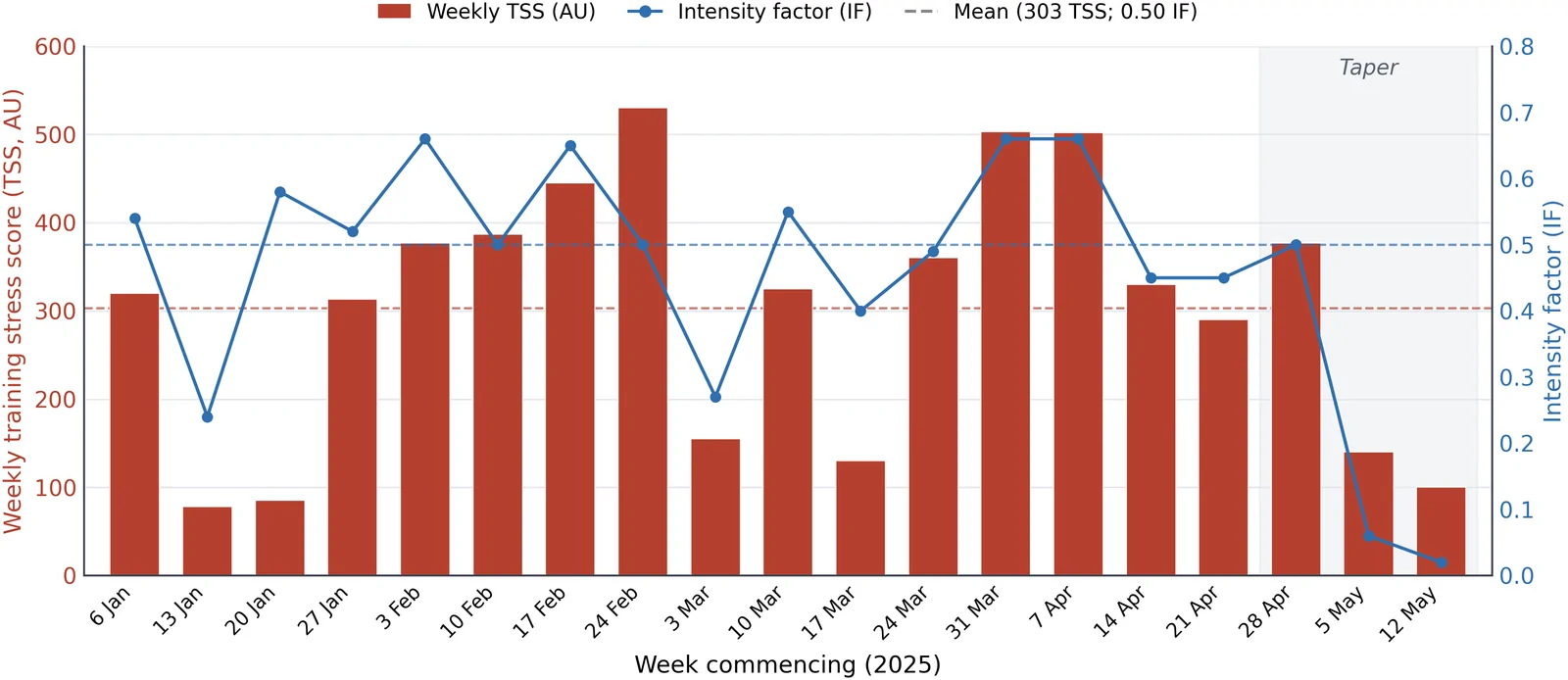
Weekly training stress score (TSS, AU; bars, left axis) and intensity factor (IF; line, right axis) across the preparation period (6 January–12 May 2025), pooled across all session types. Dashed lines denote period means (303 TSS; 0.50 IF). The shaded region indicates the two-week pre-departure taper.

A key distinguishing element of this protocol was the early initiation of low-flow supplemental O_2_ beginning at EBC (5,360 m) at a flow rate of 0.5 L/min substantially below standard expedition practice and the 2.25 L/min minimum calculated by Tannheimer and Lechner (2020) to maintain PAO_2_ >50 mmHg at the summit. This micro-dose strategy was intended to reduce AMS risk during the critical first 24 h at altitude in the complete absence of mountain acclimatization. Flow rates were increased incrementally at each camp with SpO_2_ and LLS control, reaching 3.0 L/min during the summit push consistent with approximately 35% of climbers on the mountain that season. The goal of the supplemental oxygen was to maintain the SpO_2_ during all the climb above 70%. Dexamethasone and acetazolamide were carried as emergency medication but were not self-administered.

HR and GPS were recorded by wrist device (Garmin, USA) and spot fingertip pulse oximetry (Choicemmed MD 300 C318, Beijing Choice Electronic Tech Co., Ltd, Beijing, People’s Republic of China) at each camp at rest after a 1-min stabilization period, with the subject sheltered and stationary to limit the influence of cold-induced peripheral vasoconstriction and movement artefact. A single stable spot value was recorded at each checkpoint. AMS was self-assessed at each camp using the 2018 revised LLS (Roach et al., 2018). Core temperature was additionally monitored via an ingestible telemetric pill. All biometric data were transmitted to the remote support team. Vertical ascent rate was calculated from GPS track data.

## Results

After the 18-week training program and before the start of the expedition (14 May 2025), his body mass had decreased to 101.5 kg (body fat 19.0%, skeletal muscle mass 46.8 kg), representing a 16.6 kg total mass reduction, predominantly from fat mass (body fat 22.5% to 19.0%), with a concurrent 5.5 kg (10.5%) reduction in skeletal muscle mass. Full anthropometric and fitness data across the training cycle are presented in **Table 1**. Maximal aerobic capacity (VO_2_max) was measured on 5 January 2025 at 40.8 ml·kg^−1^·min^−1^ (4.68 L·min^−1^), with an anaerobic threshold (AT) at 35.1 ml·kg^−1^·min^−1^ (86% VO_2_max) and HR at AT of 151 bpm. By 10 May 2025 (pre-expedition), VO_2_max had increased to 51.0 ml·kg^−1^·min^−1^ (5.18 L·min^−1^) and maximal HR reached 178 bpm, with HR at AT of 156 bpm representing a 25% relative improvement in VO_2_max over 18-weeks **Table 1**. In absolute terms, however, VO_2_max rose from 4.68 to 5.18 L·min^−1^ (+10.7%), the larger relative gain therefore partly reflects the concurrent 16.6 kg reduction in body mass rather than aerobic adaptation alone. Total training volume for the January–May 2025 preparation period was approximately 210–220 h, with a running distance of ~190–210 km and peak Chronic Training Load (CTL) reaching ≈50 AU (+150%). Notably, the highest-volume week (16 h, 10 March-**Figure 2**) coincided with only moderate load (325 TSS). Training was tapered in the two weeks preceding departure, reducing CTL to 40 AU and ATL to 16 AU, yielding a Training Stress Balance (TSB) of +22 AU indicating a state of peak readiness. The details of the activity (running/strength training/StairMaster), are displayed in **Figure 1**. During the tapering phase (28 April – 14 May 2025) training load was progressively reduced while session frequency and brief intensity stimuli were maintained. Weekly load (TSS) fell from approximately 378 in the first taper week to approximately 102 in the final week (~73%). Acute training load (ATL) decreased from 49 AU at the start of the taper to 16 AU and form (TSB = CTL− ATL) rose from−3 to +22, indicating attainment of peak freshness while chronic fitness (CTL) was largely preserved. Sessions remained predominantly low-intensity aerobic (heart-rate zone 1–2, 115–125 bpm), with short neuromuscular stimuli retained (5 × 50 m strides; brief zone-5/zone-2 intervals) and continued strength, mobility, core, and sport-specific work (stair climbing, inclined treadmill) to preserve climbing-specific capacity.

**Table 1:** Physical fitness and body composition across the training cycle.

Variable	Start of training cycle	Pre-expedition
Body mass (kg)	118.1	101.5
Skeletal muscle mass (kg)	52.3	46.8
Body fat (%)	22.5	19.0
VO_2_max (ml·kg^−1^·min^−1^)	40.8	51.0
VO_2_max (L·min^−1^)	4.68	5.18
Maximal HR (bpm)	167	178
Chronic Training Load, CTL (AU)	20	40
Acute Training Load, ATL (AU)	21	16
Training Stress Balance, TSB (AU)	+8	+22

Over the 18-week intensive phase, the subject completed 48 hypoxic sleep nights totaling approximately 377 h of NH sleep exposure at simulated altitudes of 1,850–5,486 m (FiO_2_ 16.7–10.5%) (**Table 2**). The cumulative hypoxic sleep dose was 4,353%·h. In addition, 16 hypoxic exercise sessions totaling 34 h were completed using a mask delivery system at simulated altitudes of 3,085–6,000 m (FiO_2_ 14.3–9.8%), yielding an exercise hypoxic dose of 426%·h. Total combined hypoxic exposure was 411 h (sleep + exercise), with a total hypoxic dose of 4,779%·h. This total exposure volume exceeds the ~300 h NH sleep estimated for prior commercial rapid ascent expeditions (Tannheimer and Lechner, 2020) and the ~290 h reported for Jornet’s preparation (Millet and Jornet, 2019) by approximately 37–42% in absolute exposure hours. Full hypoxic dose data are summarized in **Table 2**. For comparison with the altitude-based literature, the subject’s cumulative sleep exposure corresponded to approximately 1,668 km·h, within the range of documented altitude-training exposures.

**Table 2:** Summary of the 18-week normobaric hypoxia pre-acclimatization programme.

Parameter	Value
HYPOXIC SLEEP	
Simulated altitude range	1,850–5,486 m
Number of nights	48 nights
Hypoxic sleep volume	377 h
Total hypoxic sleep dose	4,353%·h
HYPOXIC EXERCISE	
Hypoxic exercise sessions	16 sessions
Hypoxic exercise volume	34 h
Total hypoxic exercise dose	426%·h
COMBINED TOTAL	
Total hypoxic exposure	411 h
Total hypoxic dose	4,779%·h

The subject departed New York (JFK) at 00:30 local time (EDT; 10:15 NST) on 15 May 2025. After a transit through Istanbul, he arrived in Kathmandu at 06:15 NST on 16 May and transferred to EBC (5,360 m) by helicopter, arriving at 11:30 NST. At EBC arrival, resting SpO_2_ was 88% on room air (HR 62 bpm; HRV 33 ms) (**Table 3**). The summit push commenced from EBC at 21:00 NST on 16 May with no rest days and no mountain acclimatization. Supplemental oxygen was initiated at EBC departure (5,360 m, 21:00 NST) at 0.5 L·min^−1^ and titrated incrementally. The subject arrived at Camp 1 (6,050 m) at 03:35 NST on 17 May (O_2_ 2.0 L/min, SpO_2_ 85%), then continued to Camp 2 (6,750 m) at 09:00 NST on 17 May (O_2_ 1.0 L/min, SpO_2_ 75%, HR 100 bpm) and reached Camp 3 (7,100 m) at 22:00 NST. At Camp 3 on the morning of 18 May (06:15 NST), SpO_2_ was 75% and HR was 95 bpm (O_2_ 1.5 L/min). He reached Camp 4 (South Col, 7,950 m) at 18:00 NST on 18 May with SpO_2_ 70% and HR 105 bpm (O_2_ 2.0 L/min). The summit push from Camp 4 commenced at approximately 00:00 NST on 19 May. The subject reached the summit of Mount Everest (8,848 m) at 09:22 NST on 19 May 2025, recording SpO_2_ 70% and HR 105 bpm (O_2_ 3.0 L/min). The sea-to-summit elapsed time was 3 days, 23 hours and 7 minutes. The ascent from EBC to summit took 60 hours and 22 minutes, corresponding to a mean ascent rate of approximately 58 m·h^−1^ approximately 10–15 times higher than conventional expeditions. Full chronological data are presented in **Table 3**. On descent, SpO_2_ recovered progressively: 80% at 7950 m (HR 100 bpm, O_2_ 3.0 L/min) and 85% at Camp 2 descent (6,750 m, HR 110 bpm, O_2_ 2.0 L/min). On return to EBC on 20 May (13:00 NST), supplemental oxygen was discontinued; SpO_2_ was 85% on room air (HR 62 bpm). He departed by helicopter to Kathmandu at 16:30 NST.

**Table 3:** Expedition chronology with physiological and symptom data during the ascent and descent.

Date	Exp. day	Altitude (m a.s.l.)	Event/stage	Time (NST)	O_2_ (L·min^−1^)	HR (bpm)	HRV (ms)	SpO_2_ (%)	LLS score	RPE
15 May	Pre	—	Departure (New York to EBC)	10:15	—	53	31	98	—	—
16 May	1	5360	EBC arrival	11:22	0	62	33	88	1	—
16 May	1	5360	EBC start of the climb	21:00	0.5	—	—	—	—	—
16–17 May	1	6,050 m	Camp 1 arrival	03:35	2	—	—	85	1	3
17 May	2	6750	Camp 2 arrival	09:45	1	100	—	75	1	3
18 May	3	7100	Camp 3 arrival	06:15	1.5	95	—	75	0	5
18 May	4	7950	Camp 4 arrival	16:20	2	105	—	70	1	6
19 May	5	8848	SUMMIT REACHED ★	09:22	3	105	—	70	—	—
19 May	5	7950	Camp 4 (descent)	—	3	100	—	80	3	7
19 May	5	6750	Camp 2 (descent)	—	2	110	—	85	3	8
20 May	6	5360	EBC return	13:07	0	62	34	85	2	5

**Table 4:** Lake Louise score components and AMS diagnosis at each expedition checkpoint.

EXPEDITION CHECKPOINT				LAKE LOUISE SCORE (LLS) — COMPONENT SCORES				TOTAL	AMS†
Date	Exp. day	Altitude (m a.s.l.)	Event/time (NST)	Head-ache	GI	Fatigue	Dizziness	LLS (0–12)	AMS diagnosis
16 May	1	5,360	EBC arrival 11:22	0	0	0	1	1	No
16–17 May	1	6,050	Camp 1 03:35	1	0	0	0	1	No
17 May	2	6,750	Camp 2 09:45	1	0	0	0	1	No
18 May	3	7,100	Camp 3 06:15	0	0	0	0	0	No
18 May	4	7,950	Camp 4 16:20	1	0	0	0	1	No
19 May	5	8,848	*Summit 09:22*	—	—	—	—	—	—
19 May	5	~8,000	Descent (upper)	1	0	2	0	3	Yes (mild) **†**
19 May	5	6,750	Camp 2 (descent)	1	0	2	0	3	Yes (mild) **†**
20 May	6	5,360	EBC return 13:07	0	0	2	0	2	No
*Mean (n = 8)*								**1,6**	

† AMS diagnosis based on LLS 2018 version.

Post-expedition sleep architecture and autonomic data are summarized in **Table 5**. Post summit recovery phase (R) followed a biphasic pattern, with a pronounced rebound on Day R + 2 followed by transient disruption on Days R + 3 and R + 4, and normalization by Day R + 5. On Day R + 1 (21 May), the subject attended a business meeting in Dubai less than 53 hours after summiting, consistent with a Garmin stress score of 22/100 and Body Battery^®^ reaching 90/100 despite travel. On Day R + 2 (22 May), a striking recovery rebound was observed: total sleep duration was 10.9 h (recovery hypersomnia), with a REM proportion of 44% (4.80 h) approximately twice the normative adult value of 20–25% consistent with REM rebound following prolonged sleep deprivation and extreme physiological stress. Resting HR fell to 47 bpm and HRV rose to 47 ms, exceeding the pre-expedition baseline of 31 ms by 52% and representing the highest HRV value recorded throughout the monitoring period. Garmin stress reached its lowest value of the recovery period (20/100) and Body Battery ^®^ peaked at 100/100, collectively indicating rapid and robust parasympathetic recovery. Days R + 3 and R + 4 showed fragmented sleep (3.77 h and 4.97 h, respectively), likely attributable to transmeridian travel and circadian disruption following the Dubai stopover rather than sustained physiological decompensation, as resting HR remained stable (49–57 bpm) and stress scores did not substantially increase. By Day R + 5, sleep duration had normalized to 7.27 h with a physiological sleep architecture (deep sleep 1.23 h, REM 1.28 h), consistent with return to baseline function. Full overview of intervention period is outlined in **Table 6**. The rapid autonomic recovery observed here, with HRV returning to and exceeding baseline by Day R + 2 (based on the two available recovery-day readings), is consistent with the well-trained cardiovascular profile documented pre-expedition and may reflect a favorable interaction between the chronic hypoxic stimulus of the NH program and the acute physiological response to extreme-altitude exercise. Notably, this recovery trajectory occurred in the context of intercontinental air travel and professional obligations, underscoring the real-world feasibility of the approach beyond the physiological outcome alone.

**Table 5:** Post-expedition sleep architecture and autonomic recovery.

Date	Recovery day	Total sleep (h)	Deep sleep (h)	REM sleep (h)	Light sleep (h)	Resting HR (bpm)	HRV (ms)	Garmin stress
21 May	R+1	7.2	1.15	2.02	4.03	51	34	22
22 May	R+2	10.9	0.98	4.8	5.13	47	47	20
23 May	R+3	3.77	0.98	0.43	2.35	49	—	27
24 May	R+4	4.97	1.17	0.6	3.2	57	—	34
25 May	R+5	7.27	1.23	1.28	4.75	53	—	25

**Table 6:** Overview of the intervention period, training phases, assessment points, and measured outcomes.

Phase/period	Dates (2025)	Training focus	Normobaric-hypoxia (NH) exposure	Assessments & measured outcomes
Baseline assessment	5–6 Jan	—	—	VO_2_max (incremental treadmill, breath-by-breath); body composition; 12-lead ECG, echocardiography, maximal stress test; full blood panel incl. iron status
Build phase (Wk 1–7)	6 Jan – 23 Feb	Aerobic base, running economy, strength, StairMaster-specific endurance	NH sleep at low–moderate simulated altitude; first mask-based hypoxic exercise sessions	Weekly TSS, IF, CTL/ATL/TSB; nightly SpO_2_/HR; cumulative hypoxic dose (%·h)
Peak-load block	24 Feb – early Apr	Highest training load (peak 530 TSS); combined volume and intensity	NH sleep progressing toward 5,486 m; hypoxic exercise toward 6,000 m (16 sessions over the cycle)	Load metrics; nightly SpO_2_/HR; hypoxic-dose accrual
Recovery weeks	early–mid Mar	Reduced load (130–155 TSS)	Continued NH sleep	Load metrics; SpO_2_/HR
Taper (Wk 17–18)	28 Apr – 14 May	Load reduced ~73%; intensity stimuli and neuromuscular work retained	Tapered NH exposure	CTL 40, ATL 16, TSB + 22
Pre-expedition assessment	10 May	—	—	VO_2_max retest; body composition; blood panel
Expedition (sea-to-summit)	15–20 May	Ascent EBC to summit, without acclimatization rotations	Hypobaric hypoxia and staged, titrated supplemental O_2_ (0.5–3.0 L·min^−1^)	Camp-by-camp SpO_2_, HR, HRV, LLS, O_2_ flow, core temperature, RPE; GPS time/altitude
Post-expedition recovery	21–25 May (R + 1–R+5)	—	—	Sleep architecture, HRV, resting HR, Garmin stress (descriptive wearable data)

## Discussion

This case report documents a rapid sea-to-summit ascent of Mount Everest (3 days 23 h 7 min) by a non-elite, non-professional individual, completed without on-mountain acclimatization rotations and without pharmacological or inert-gas (xenon) pre-acclimatization aids, while using conventional supplemental oxygen and professional Sherpa support. The availability of objective pre-acclimatization data including VO_2_max progression, body composition, training load metrics, daily hypoxic dose records, and camp-by-camp SpO_2_/HR makes this one of the most comprehensively documented rapid Everest ascents in the literature.

The 25% improvement in VO_2_max (from 40.8 to 51.0 ml·kg^−1^·min^−1^) over 18 weeks is substantial and reflects the combined effect of intensified normoxic training (peak CTL 50 AU, peak weekly TSS 530) and hypoxic training adaptation. While the subject’s pre-training VO_2_max of 40.8 ml·kg^−1^·min^−1^ is below published values for elite high-altitude climbers (Oelz et al., 1986), the final value of 51.0 ml·kg^−1^·min^−1^ approaches those of trained recreational endurance athletes and may have been sufficient for the demands of the ascent when combined with NH pre-acclimatization and supplemental O_2_. The 16.6 kg reduction in body mass (particularly the 27.4% decrease in fat mass) likely contributed meaningfully to the improved relative VO_2_max and reduced mechanical cost of ascent. This relative gain should nonetheless be interpreted with caution: in absolute terms VO_2_max increased only from 4.68 to 5.18 L·min^−1^ (+10.7%).

The NH program comprised 48 sleep nights (377 h; simulated altitudes 1,850–5,486 m) and 16 hypoxic exercise sessions (34 h), yielding a total hypoxic exposure of 411 h and a cumulative hypoxic dose of 4,779%·h (4,353%·h sleep + 426%·h exercise). This total exposure volume exceeds published precedents by approximately 37–42%: Tannheimer and Lechner (2020) estimated ~300 h NH sleep for commercial rapid expeditions, and Millet and Jornet (2019) reported ~260 h sleep plus ~30 h hypoxic exercise (~290 h total) in preparation for Jornet’s world-record ascent. The present program distinguished itself not only in total volume but in the progressive elevation of simulated altitude, reaching 5,486 m in the final sessions, a ceiling substantially above the 3,500–4,500 m commonly reported in the literature.

Using the 2018 Lake Louise Score (Roach et al., 2018), no AMS was diagnosed during the ascent phase. LLS was 0–1 at EBC, Camp 1, Camp 2, Camp 3, and Camp 4 despite a completely unacclimatized approach and an ascent rate (~58 m·h^−1^) substantially exceeding the pace maintained by professional high-altitude guides (**Table 4**). The LLS was not formally assessed at the summit owing to conditions; the absence of diagnosed AMS therefore applies up to Camp 4 (7,950 m), not to the summit. The maintenance of LLS 0–1 across the entire assessed ascent phase, despite a completely unacclimatized approach, is clinically remarkable. Zhao et al. (2024) documented AMS rates of 37.9% at only 3,650 m in a rapid-ascent cohort arriving by airplane at a fraction of the altitude reached by the present subject and under conditions far less physiologically extreme. Mild AMS (LLS = 3; Headache 1 + Fatigue 2) was recorded on the descent at Camp 4 (7,950 m) and at Camp 2 (6,750 m). AMS criteria were no longer met by EBC return (LLS = 2; Fatigue 2, no headache), though residual fatigue persisted. The onset on descent rather than during ascent is of clinical interest. At this stage, the subject had been continuously above 7,000 m for approximately 40 hours with no rest, completing the highest-altitude segment and partial descent at an intensity exceeding the operational limits of most acclimatized professional climbers. The LLS Fatigue item (grade 2, “moderately fatigued, reduced motivation, low energy”) is substantially confounded by exertional exhaustion and sleep deprivation at this stage, making clean attribution to altitude-specific pathophysiology difficult. Nonetheless, the co-occurrence of grade 1 headache satisfies formal AMS criteria, and mild AMS on descent cannot be excluded. Crucially, no HACE or HAPE developed, and full symptom resolution occurred within hours of descent to EBC a pattern more consistent with exertional fatigue superimposed on mild hypoxic stress than with progressive altitude illness. The present data therefore suggest that the combined NH pre-acclimatization and graded supplemental O_2_ strategy was associated with the absence of diagnosed AMS in this individual throughout the ascent, with mild AMS criteria met only on the descent after an unprecedented sustained effort. This distinction — an AMS-free ascent with mild AMS on descent — is itself a novel clinical finding in the context of a record-pace unacclimatized Everest attempt.

The SpO_2_ values recorded at key waypoints provide the first objective in-ascent physiological dataset for a sea-to-summit rapid ascent without on-mountain acclimatization. At Camp 2 (6,750 m, O_2_ 1.0 L/min), SpO_2_ was 75% with HR 100 bpm. SpO_2_ of 75% at Camp 3 (7,100 m, O_2_ 1.5 L/min, HR 95 bpm) is consistent with published values for acclimatized climbers at similar altitudes on low-flow supplemental oxygen (Tannheimer and Lechner, 2020), suggesting the NH pre-acclimatization produced adequate acclimatization equivalence across the 6,750–7,100 m range. SpO_2_ of 70% at Camp 4 arrival (7,950 m, O_2_ 2.0 L/min, HR 105 bpm) and at the summit (8,848 m, O_2_ 3.0 L/min, HR 105 bpm) falls within the range expected for climbers on standard supplemental O_2_ flows at these altitudes (Tannheimer and Lechner, 2020). The equivalent SpO_2_ at both camps despite a 900 m altitude gain and an increase in O_2_ flow from 2.0 to 3.0 L/min reflects appropriate titration of supplemental oxygen to maintain a consistent physiological corridor. The consistent HR of 105 bpm at both Camp 4 and summit reflects a stable cardiovascular response despite extreme altitude, consistent with maintained aerobic performance capacity in the death zone. During descent, SpO_2_ recovered progressively from 70% at summit to 80% at 7,950 m (O_2_ 3.0 L/min, HR 100 bpm) and to 85% at Camp 2 descent (6,750 m, O_2_ 2.0 L/min, HR 110 bpm). On return to EBC, supplemental oxygen was discontinued; SpO_2_ was 85% on room air (HR 62 bpm, HRV 34 ms) equivalent to pre-departure baseline confirming return to physiological stability within approximately 28 hours of summiting. The HR of 62 bpm at EBC return matches the subject’s resting HR recorded at EBC arrival before the climb, indicating full cardiovascular recovery rather than residual physiological stress.

The early low-flow O_2_ approach (0.5 L/min from EBC) is novel. Tannheimer and Lechner (2020) calculated that 2 L/min at rest is sufficient to maintain PAO_2_ above 50 mmHg even at the summit, and that 2.25 L/min is the summit minimum at rest. The 0.5 L/min used at lower camps falls below physiologically significant thresholds for O_2_ enrichment at 5,000–7,000 m in an acclimatized individual, yet may have provided a meaningful safety margin in the completely unacclimatized state during the critical first 48 h. LLS remained 0–1 at Camps 2 and 3, altitudes far above the 3,650 m at which Zhao et al. (2024) report an AMS prevalence of 37.9% in rapid-ascent cohorts. This is consistent with an effect of the combined approach, although the contribution of each component cannot be separated.

Compared to Jornet’s preparation (Millet and Jornet, 2019), the current subject had a lower baseline VO_2_max (40.8 vs. ~92 ml·kg^−1^·min^−1^), higher body mass (118 vs. 56 kg), and no dedicated alpine HH phase. The present sea-to-summit time (3 days 23 h from New York) is not directly comparable with Jornet's fastest known time (26.5 h from Rongbuk monastery at 5,100 m), which measures the mountain segment only; the door-to-summit metric reported here is the more relevant one for non-professional climbers with professional and family commitments. The higher NH hours (411 h vs. ~290 h for Jornet) and the early O_2_ strategy appear to have compensated for the inferior baseline fitness and absence of an on-mountain HH adaptation phase. The ascent rate from EBC to summit (~58 m·h^−1^) was 10–15 times higher than typical conventional expedition rates, consistent with adequate physiological performance at extreme altitude in this individual; causation cannot be established.

## Subject perspective

The perspective of the subject on this attempt extends well beyond the physiological record itself. The original motivation was pragmatic rather than competitive: after climbing Denali which took 20 days including the commute from New York the prospect of being absent from family and work for the 50 days traditionally required to acclimatize for Everest was simply not feasible. The idea was first to test the method on a lower mountain Aconcagua, which was climbed in four days including travel from New York before receiving clearance to attempt Everest. The 18-week preparation program in the subject's account, as mentally demanding as the ascent itself. Sleeping for months in a hypoxic tent, long workouts, dietary discipline, professional and family pressures, the psychological weight of the 2024 failure, and the awareness that no one had ever climbed Everest in so few days without acclimatization collectively represented a sustained mental load that required as much management as the physiological conditioning. Regarding the broader significance of the attempt, the project was described not primarily as a record attempt but as a demonstration showing a way forward, not setting a ceiling. The decision to train exclusively with NH explicitly avoiding substances such as dexamethasone or xenon gas, which he considers unethical and potentially dangerous reflects a deliberate commitment to WADA-compliant methods and to demonstrating what is achievable through preparation alone. The strength of this case is precisely this combination: a non-elite, non-professional individual with measurable physiological limitations, full transparency of preparation data, and a record outcome achieved under strictly conventional pharmacological conditions. As the subject noted, the technology used hypoxic tents and supplemental oxygen is more than 20 years old and broadly accessible. What this case demonstrates is that systematic application of existing methods, rigorously individualized and medically supervised, can produce outcomes that the mountaineering community had considered impossible.

From a practical standpoint, this case illustrates how existing, broadly accessible technologies: overnight normobaric-hypoxic tents, mask-based hypoxic exercise, consumer wearables, and supplemental oxygen can be integrated into a structured, home-based preparation that fits around professional and family commitments rather than requiring prolonged residence at altitude. For clinicians, coaches, and expedition physicians, the most readily transferable elements are the underlying principles rather than the specific outcome: quantification of the hypoxic stimulus as a cumulative dose (%·h) delivered through scheduled sleep and exercise exposure; objective monitoring of training load and freshness (TSS, CTL, ATL, TSB) to time a pre-departure taper; staged, individually titrated low-flow supplemental oxygen under SpO_2_ and symptom control and continuous medical supervision with predefined safety thresholds. Applied together, these principles offer a framework for planning and individualizing time-efficient pre-acclimatization and for generating the kind of objective dataset that future research will require. At the same time, the present results should not be interpreted as a validated protocol: the favorable outcome reflects an integrated strategy in a single, highly selected, and closely supervised individual, and the independent contribution of normobaric hypoxia cannot be isolated from concurrent training, body-mass reduction, staged supplemental oxygen, expert guiding, and individual hypoxia tolerance. We therefore caution strongly against unsupervised replication and emphasize the need for controlled prospective studies to define the minimum effective hypoxic dose, the safety envelope, and the independent role of each component before strategies of this kind can be recommended for wider practice.

## Practical implementation

The principles illustrated by this case can be operationalized as a stepwise, individualized framework, while recognizing that a single favorable outcome does not validate the protocol. First, candidates undergo baseline medical and performance screening (cardiac evaluation, blood and iron status, VO_2_max, body composition) to establish safety and individualized targets. Second, the hypoxic stimulus is prescribed as a cumulative dose target (%·h) delivered through scheduled overnight NH sleep and mask-based hypoxic exercise, beginning at low simulated altitude and progressing as tolerated under nightly SpO_2_ and HR monitoring. Third, training load and freshness are tracked objectively (TSS, CTL, ATL, TSB) to build aerobic and climbing-specific capacity and to time a pre-departure taper that restores freshness while preserving fitness. Fourth, on the mountain, supplemental oxygen is introduced in a staged, individually titrated manner under continuous SpO_2_ and LLS control, with predefined flow-adjustment and abort thresholds. Throughout, continuous medical supervision and explicit safety criteria are maintained. This framework is intended to support the planning and individualization of time-efficient pre-acclimatization and to generate the objective datasets that future controlled studies will require; it is explicitly not a recommendation for unsupervised replication.

## Limitations

This case report documents a rapid sea-to-summit ascent of Mount Everest (3 days 23 h 7 min) by a non-elite, non-professional individual, completed without acclimatization rotations and without pharmacological or inert-gas (xenon) pre-acclimatization aids. Accordingly, the outcome reflects an integrated preparation-and-ascent strategy rather than any single factor (early O_2_, Sherpa support, individual resilience). In the absence of a control condition, it cannot be attributed solely to normobaric-hypoxia pre-acclimatization. While the expedition chronology and summit were externally corroborated (GPS data, Elite Exped and Sherpa-team records, and the official Nepalese summit certificate), the pre-expedition physiological testing, the hypoxic-exposure logs, and the in-ascent SpO_2_, heart-rate, and Lake Louise Score data were acquired and interpreted by the corresponding author without independent third-party verification. Independent measurement or audit of these data would strengthen confidence in the reported values. No direct markers of the candidate adaptive mechanisms were obtained: total hemoglobin mass, erythropoietin, hypoxic ventilatory response, arterial blood gases, and pulmonary-function and mitochondrial markers were not measured. Any attribution of the outcome to hematological, ventilatory, or mitochondrial adaptation therefore remains speculative and hypothesis-generating, to be tested in controlled prospective studies.

Within these constraints, the case provides a comprehensive physiological characterization of a rapid Everest ascent and is consistent with the feasibility of NH pre-acclimatization as part of a time-efficient strategy, without establishing that it can replace on-mountain acclimatization. The absence of a control condition prevents attribution of outcomes solely to NH pre-acclimatization versus other factors. The author strongly cautions against attempts to replicate this protocol without equivalent preparation, expert medical supervision, and safety infrastructure.

## Conclusion

In conclusion, this case report documents, to our knowledge, the fastest documented sea-to-summit ascent of Mount Everest (3 days 23 h 7 min) by a non-professional individual, achieved without on-mountain acclimatization rotations and without pharmacological or inert gas (xenon) aids. This case may contribute to the growing body of evidence that may inform future controlled study of NH pre-acclimatization as a time-efficient alternative to traditional on-mountain preparation for non-elite individuals undertaking extreme-altitude ascents. A 25% gain in VO_2_max (40.8 to 51.0 ml·kg^−1^·min^−1^), a cumulative hypoxic dose of 4,779%·h over 48 sleep nights and 16 exercise sessions, and a peak CTL of 50 AU provide one of the most comprehensive physiological characterizations of a rapid Everest ascent published to date, enabling direct comparison with future studies.

No AMS was diagnosed during the assessed ascent (LLS 0–1 up to Camp 4, 7,950 m); only mild AMS (LLS = 3) appeared on descent, resolving by EBC return. Together with the SpO_2_ data, this suggests that a structured high-dose NH program, combined with early low-flow supplemental O_2_ and expert medical supervision, may be feasible as a time-efficient preparation strategy in a well-prepared, closely supervised non-elite individual; its independent contribution cannot be isolated from supplemental oxygen, Sherpa support, training, and body-mass reduction. 70% at Camp 4 and summit on 3.0 L/min O2 confirm maintained oxygenation within the targeted range at extreme altitude.

Priority research questions include: (1) what is the minimum effective NH hypoxic dose (%·h) for safe acclimatization-free Everest ascent; (2) what hematological and ventilatory adaptations underlie the present outcomes; and (3) whether early micro-dose supplemental O_2_ provides independent AMS protection beyond NH pre-acclimatization alone. Controlled prospective studies in non-elite subjects are strongly recommended.

## Data Availability

The raw data supporting the conclusions of this article will be made available by the authors, without undue reservation.
